# The Transepithelial Transport and Transport Pathways of Free and Bound Nε-Carboxymethyllysine Using a Caco-2 Cell Monolayer Model

**DOI:** 10.3390/foods15142432

**Published:** 2026-07-09

**Authors:** Xiaojin Yuan, Chenxi Nie, Ruohan Zhai, Aobai Tu, Xinyu Cao, Li Zhang, Juxiu Li

**Affiliations:** 1 College of Food Science and Engineering, Gansu Agricultural University, Lanzhou 730070, China; 2 College of Food Science and Engineering, Northwest A&F University, Yangling 712100, China

**Keywords:** advanced glycation end products, Nε-carboxymethyllysine, transport rate, transport pathway, Caco-2 cell monolayers

## Abstract

As a typical marker of dietary advanced glycation end products (AGEs), Nε-carboxymethyllysine (CML) exists in both free and protein-bound forms in foods, exhibiting distinct intestinal transport behaviors. This study aimed to characterize the transepithelial transport, intracellular accumulation and transport pathways of free CML (FC) and protein-bound CML (BC, BSA-CML) using Caco-2 cell monolayers. Since protein-bound CML undergoes digestion in the gastrointestinal tract, BC was subjected to simulated gastrointestinal digestion before transport experiments to better mimic human digestion (BC digests). The results showed that FC exhibited significantly greater transepithelial transport across Caco-2 cell monolayers than BC digests. The transport rate of CML from FC across Caco-2 cell monolayers was 13.04%, significantly higher than that of BC digests (0.99%). Consistently, the intracellular accumulation of total CML was higher for FC (2.62%) than for BC digests (0.14%). Furthermore, FC transport appeared to occur mainly via simple diffusion, whereas the transepithelial transport of CML derived from BC digests likely involved a peptide transporter 1 (PepT1)-associated transport pathway. These findings provide insights into the transepithelial transport pathways and intracellular accumulation of dietary free and bound AGEs.

## 1. Introduction

Advanced glycation end products (AGEs) are a heterogeneous group of compounds generated by non-enzymatic glycation reactions between reducing sugars and the amino groups of amino acids or proteins [1]. These reactions occur naturally in biological systems at physiological temperature (37 °C), leading to the gradual formation of endogenous AGEs through interactions between carbohydrates and proteins over extended periods [2]. In addition to endogenous formation, AGEs are widely generated during food processing and cooking, particularly in processed nuts, baked goods, and fried meats, and are therefore referred to as exogenous AGEs [3]. Together, endogenous and dietary AGEs constitute the total body AGE burden, with dietary intake representing the major source of exogenous AGEs [4]. Moreover, recent studies have demonstrated that AGEs can also be generated during the simulated gastrointestinal digestion of meal-resembling systems [5] and α-dicarbonyl–whey protein mixtures [6], indicating that gastrointestinal formation may represent an additional in vivo source of AGEs. Accumulating evidence has linked AGEs to the development and progression of metabolic diseases, including obesity, diabetes, dyslipidemia, atherosclerosis, and alcoholic fatty liver disease [7]. These pathological effects are believed to arise, at least partly, from the AGE-induced activation of the receptor for advanced glycation end products (RAGE), which subsequently promotes oxidative stress and inflammatory signaling pathways [8,9]. Given that dietary AGEs may cross the intestinal epithelial barrier and enter the circulation, understanding their intestinal transport behavior is essential for clarifying their potential contribution to the development of metabolic diseases.

AGEs in foods and biological systems exist in two forms: free AGEs, which are generated through reactions between reducing sugars and free amino acids, and protein- bound AGEs, which are formed through the glycation of proteins [10,11]. Soy sauce and beer are rich in free AGEs, whereas meat and meat products, milk, and dairy products predominantly contain bound AGEs [12]. Previous studies extensively investigated changes in the concentration of free and bound AGEs during processing [10,11,13,14]. This distinction is biologically significant because free and protein-bound AGEs exert different effects on hepatic glucose metabolism [15], gut microbiota composition [16], and the activation of Nrf2 and NF-κB signaling pathways [17]. Among various AGE compounds, Nε-carboxymethyllysine (CML) is widely recognized as a representative marker of dietary AGEs and is frequently used to assess the AGE content of foods [18]. In our previous study, pharmacokinetic analysis revealed the substantially higher absorption of free CML (FC) than bovine serum albumin (BSA)–CML (BC) in rats [19]. However, the reasons underlying this difference remain unclear. We hypothesized that the disparity in absorption may be partially attributed to differences in intestinal transport pathways.

Compounds can cross the intestinal epithelium through four major routes: passive diffusion, paracellular transport, transcytosis, and carrier-associated transport [20]. Because chemically stable AGEs such as CML largely resist degradation during digestion, they can be transported directly into intestinal epithelial cells [21]. Hellwig et al. [22] reported that FC exhibited low affinity for the intestinal lysine transporter during transport across Caco-2 cell monolayers and hypothesized that FC uptake occurs predominantly via simple diffusion rather than active transport. Nevertheless, direct experimental evidence supporting this transport pathway remains limited and warrants further investigation. In contrast, protein-bound AGEs must first undergo gastrointestinal digestion, thereby generating free and peptide-bound AGEs that are subsequently available for absorption. A previous study demonstrated that digested myofibrillar protein-bound CML crossed Caco-2 cell monolayers through multiple pathways, including paracellular transport, transcytosis, and PepT1-associated transport [23]. These findings suggest that the chemical form of CML may fundamentally influence its intestinal transport behavior. Despite these observations, existing studies have examined either free CML [22] or myofibrillar protein-bound CML [23] independently, and a direct comparison under identical experimental conditions is still lacking. Such a comparison is essential because free and protein-bound AGEs coexist in the human diet and may differ substantially in both absorption efficiency and transport pathways. Furthermore, the structural diversity of glycated proteins can influence digestion patterns and generate distinct peptide-bound CML species, which may further affect transport rates and pathways.

Although the Caco-2 cell monolayer model has limitations, including the absence of mucus, digestive dynamics, gut microbiota interactions, and systemic metabolism, it remains one of the most widely accepted in vitro models for evaluating intestinal epithelial transport because of its simplicity, reproducibility, reliability, and cost-effectiveness compared with in vivo animal studies [24]. Therefore, Caco-2 cell monolayers were employed in the present study to investigate the transepithelial transport and transport pathways of FC and BC digests, aiming to explain the pharmacokinetic differences previously observed in vivo. Specifically, we hypothesized that (1) CML derived from FC may exhibit a higher transport rate than CML derived from BC digests, and (2) this difference is primarily driven by distinct transport pathways, whereby FC may be transported mainly via simple diffusion, whereas CML derived from BC digests may be transported through paracellular, transcytosis, and PepT1-associated pathways. Unlike previous studies that focused on a single AGE form or a single transport parameter, the present work simultaneously evaluated bidirectional transport, intracellular accumulation, and transport pathways, thereby providing a more comprehensive understanding of how the chemical form of dietary CML governs its intestinal transport behavior.

## 2. Materials and Methods

### 2.1. Materials and Reagents

Standards of CML-d_4_ (purity > 97%) and CML (purity > 99%) were supplied by Iris Biotech GmbH (Marktredwitz, Germany). Fetal bovine serum (FBS) was obtained from ZETA Life (Menlo Park, CA, USA). Nonessential amino acids, penicillin–streptomycin solution (100×), Dulbecco’s Modified Eagle Medium (DMEM) and 0.25% trypsin–ethylenediaminetetraacetic acid (EDTA) were purchased from Gibco (Grand Island, NY, USA). Sodium fluorescein and BSA were sourced from Sigma-Aldrich (St. Louis, MO, USA). PBS and Hanks’ balanced salt solution (HBSS) were acquired from Solorio (Beijing, China). The FC (purity ≥ 95%) used in the cell experiments was procured from Qingdao Vochem Co., Ltd. (Qingdao, China). Primary antibodies against peptide transporter 1 (PepT1, Catalog No. HA500444) and GAPDH (Catalog No. ET1601-4) were purchased from HUABIO (Hangzhou, China).

### 2.2. Synthesis of BSA-CML

BSA has been widely used as a model protein in food systems because of its well-characterized structure and physicochemical properties, making it particularly suitable for investigating the digestive behavior and potential health effects of glycated proteins [15,25,26]. The synthesis of BC, together with the characterization of native BSA and the resulting BC, was performed as previously described in our earlier study [16]. In the native BSA, lysine accounted for 8.481%, whereas the lysine content decreased to 2.308% following glycation, indicating the extensive modification of lysine residues during CML formation. The CML content of the resulting BSA-CML was 7.88 g/100 g of sample.

### 2.3. In Vitro Simulated Gastrointestinal Digestion of BSA-CML

The in vitro simulated gastrointestinal digestion of BC was performed according to the method described in our previous study [27]. Briefly, 100 mg of BSA-CML was dissolved in 8 mL of 1.25× simulated gastric fluid containing NaHCO_3_, NaCl, KCl, HCl, KH_2_PO_4_, (NH_4_)_2_CO_3_, and MgCl_2_(H_2_O)_6_. Then, 0.005 mL of CaCl_2_ (H_2_O)_2_ (0.3 M) was added, and the pH was adjusted to 3 with HCl. Pepsin solution (20 mg/mL) was then added, and the final volume was adjusted to 10 mL, resulting in a pepsin activity of 2000 U/mL. Gastric digestion was conducted at 37 °C for 2 h.

Following gastric digestion, 7 mL of 1.25× simulated intestinal fluid containing NaHCO_3_, NaCl, KCl, MgCl_2_(H_2_O)_6_, KH_2_PO_4_, and HCl was added to the gastric chyme. Subsequently, 0.04 mL of CaCl_2_(H_2_O)_2_ (0.3 M) was added, and the pH was adjusted to 7 using NaOH. Pancreatin suspension was then added to obtain a final activity of 100 U/mL, and intestinal digestion was performed at 37 °C for an additional 2 h. All digestion experiments were conducted in triplicate.

Upon the completion of digestion, the resulting BC digests were lyophilized and stored at 4 °C until further use. The contents of total CML, free CML and 10% TCA-precipitated bound CML (representing CML associated with proteins or peptides > 5 kDa) were 25.95 ± 0.54, 0.69 ± 0.03 and 0.70 ± 0.02 mg/g digest, respectively. Based on these measurements, the bound CML content was calculated as the difference between total CML and free CML, yielding 25.26 mg/g. Furthermore, peptide-bound CML (defined as CML associated with peptides smaller than 5 kDa, including dipeptides and tripeptides) was calculated by subtracting both free CML and TCA-precipitable (>5 kDa) CML from total CML, resulting in a value of 24.56 mg/g [27]. Based on these calculations, most CML remained associated with low-molecular-weight peptides following gastrointestinal digestion. To minimize variability among digestion batches, the three lyophilized BC digests obtained from independent digestion experiments were pooled, thoroughly mixed and used for subsequent experiments.

### 2.4. Caco-2 Cell Culture

Caco-2 cells (passages 23–33) were cultured in DMEM supplemented with 1% nonessential amino acids, 1% (*w*/*v*) penicillin (100 U/mL)–streptomycin (100 µg/mL), and 10% (*v*/*v*) FBS in a humidified incubator containing 5% CO_2_ at 37 °C. The culture medium was replaced every other day, and cells were trypsinized with 0.25% trypsin–EDTA solution (Sigma-Aldrich, Buchs, Switzerland) when they reached 80–90% confluence. To obtain differentiated enterocyte-like cells, Caco-2 cells were cultured for 21 days in 96-well and 6-well plates or 12-well Transwell inserts. The differentiated cells cultured in 96-well and 6-well plates were used for cell viability assays and peptide transporter 1 (PepT1) expression analysis, respectively. Additionally, 12-well Transwell inserts and conventional 12-well plates were used for transepithelial transport experiments and transport pathway studies.

### 2.5. Caco-2 Cell Viability Assay

Cell viability following FC and BC digest treatments was assessed using a Cell Counting Kit-8 (CCK-8, Nanjing Jiancheng Bioengineering Institute, Nanjing, China). Caco-2 cells were seeded in 96-well plates at a density of 1 × 10^4^ cells/well and cultured for 21 days to establish differentiated Caco-2 monolayers. The culture medium was replaced every other day during the first week and daily thereafter. On day 21, the culture medium was replaced with fresh medium, and the differentiated monolayers were exposed to FC (0.01–0.12 mg/mL) or BC digests (0.5–6 mg/mL) for 24 h, while untreated cells served as the control group. In the BC digest treatment group, the corresponding CML concentration ranged from 0.0125 to 0.15 mg/mL. Each treatment was performed with six independent replicates. Following treatment, the medium was discarded, and 110 μL of freshly prepared CCK-8 working solution (100 μL complete medium plus 10 μL CCK-8 reagent) was added to each well. After incubation at 37 °C for 2 h, absorbance, which reflects the number of surviving cells, was determined at 450 nm using a microplate reader (Tecan Austria GmbH, Grodig, Austria).

### 2.6. Establishment and Evaluation of Caco-2 Cell Monolayer Model

#### 2.6.1. Establishment of Caco-2 Cell Monolayer Model

To establish the Caco-2 cell monolayer model, cells were resuspended at a density of 2 × 10^5^ cells/mL and subsequently seeded onto polyester (PET) Transwell inserts (12-well, 0.4 μm pore size, 1.12 cm^2^ growth area; Corning, Acton, MA, USA). A volume of 0.5 mL of the cell suspension was added to the apical (AP) compartment, while 1.5 mL of complete culture medium was added to the basolateral (BL) compartment. The cells were incubated in a humidified incubator containing 5% CO_2_ at 37 °C for 21 days to allow for differentiation and monolayer formation. The culture medium on both sides of the inserts was replaced every other day during the first week and daily thereafter.

#### 2.6.2. Evaluation of Caco-2 Cell Monolayer Model

##### Transepithelial Electric Resistance (TEER) Measurement

TEER values were measured to evaluate monolayer integrity. A TEER value exceeding 300 Ω·cm^2^ was considered suitable for transepithelial transport experiments [28]. The electrical resistance of Caco-2 monolayers was measured using a Millicell^®^ ERS-2 (Millipore Corporation, Bedford, MA, USA) on days 4, 8, 12, 16, 20, and 21 after seeding. TEER values were calculated as follows:

(1) TEER = (R − R_0_) × A

where R is the resistance of the insert with the cell monolayer, R_0_ is the resistance of the blank insert without cells, and A is the membrane surface area (1.12 cm^2^).

##### Alkaline Phosphatase (ALP) Activity Assay

ALP activity was measured as a marker of Caco-2 monolayer differentiation. Culture medium was collected separately from the AP and BL compartments immediately before medium replacement on days 7, 14, and 21. ALP activity was determined using a commercial ALP assay kit (Nanjing Jiancheng Bioengineering Institute, Nanjing, China) with three technical replicates, and the ratio of ALP activity in the AP compartment to that in the BL compartment was calculated.

##### Paracellular Permeability Assay

The permeability of sodium fluorescein across Caco-2 monolayers was evaluated as an indicator of paracellular permeability. The assay was performed in triplicate according to the method of Catanzaro et al. [29] with minor modifications. Briefly, 0.5 mL of HBSS containing 20 μg/mL sodium fluorescein was added to the AP compartment, while 1.5 mL of HBSS was added to the BL compartment. After incubation for 2 h, the amount of sodium fluorescein transported to the BL side was quantified using a microplate reader (Tecan Austria GmbH, Grodig, Austria) at an excitation wavelength of 460 nm and an emission wavelength of 515 nm. The concentration of sodium fluorescein was determined using a standard calibration curve. The apparent permeability coefficient (*P_app_*) was calculated using the following equation:

(2) $$P_{\mathrm{app}}\;=\;\frac{\mathrm{dQ}}{\mathrm{dt}}\times \frac{1}{A\;\times \;C_{0}}$$

where dQ/dt represents the steady-state flux (μg/s), A denotes the surface area of the monolayer membrane (cm^2^), and C_0_ signifies the initial concentration in the AP compartment (μg/mL).

### 2.7. Transepithelial Transport and Intracellular Accumulation Experiments

#### 2.7.1. Bidirectional Transport Experiments

Bidirectional transport experiments of FC and BC digests were performed in quadruplicate according to the method described by Ye et al. [23] and Qi et al. [30]. Prior to transport experiments, differentiated Caco-2 monolayers cultured on Transwell inserts were transferred to fresh 12-well plates and equilibrated with HBSS for 30 min at 37 °C in a humidified incubator containing 5% CO_2_. Specifically, 0.5 mL and 1.5 mL HBSS were added to the AP and BL compartments, respectively. Following equilibration, HBSS was removed from both compartments before initiating transport experiments.

(1) AP-to-BL transport (AP-BL): A total of 0.5 mL of HBSS containing FC or BC digests at the same CML concentration was added to the AP compartment, while 1.5 mL of fresh HBSS was added to the BL compartment. After incubation for 2 h at 37 °C, 1 mL of solution was collected from the BL compartment for CML quantification by HPLC-MS/MS. *P_app_* was calculated using Equation (2), and the transport rate was calculated according to the following equation:

(3) Transport rate_(AP-BL)_ = CML_BL side_/CML_AP side_ × 100%

where CML_BL side_ denotes the amount of CML detected in the BL compartment after transport, and CML_AP side_ denotes the initial amount of CML added to the AP compartment.

(2) BL-to-AP transport (BL-AP): A total of 1.5 mL of HBSS containing FC or BC digests at the same CML concentration was added to the BL compartment, while 0.5 mL of fresh HBSS was added to the AP compartment. Following a 2 h incubation, 0.4 mL of solution was collected from the AP compartment for HPLC-MS/MS analysis. *P_app_* and the transport rate were calculated according to Equations (2) and (4):

(4) Transport rate_(BL-AP)_ = CML_AP side_/CML_BL side_ × 100%

where CML_AP side_ denotes the amount of CML detected in the AP compartment after transport, and CML_BL side_ denotes the initial amount of CML added to the BL compartment.

To assess monolayer integrity after transport, TEER values were measured at the completion of each transport experiment, including the transport inhibitor experiments. No significant reduction in TEER values was observed, indicating that the Caco-2 monolayer remained intact at the end of the transport period.

#### 2.7.2. Intracellular Accumulation of CML

To further evaluate cellular uptake, the intracellular accumulation of CML was measured using differentiated Caco-2 monolayers following the AP-BL transport protocol described above. The procedure was adapted from Hellwig et al. [22] and Ma et al. [31], with slight modifications. After 2 h of transport, the monolayers were gently rinsed three times with PBS to remove residual extracellular CML. The cells were then harvested and resuspended in 300 μL PBS. To achieve complete cell lysis, samples were subjected to four freeze–thaw cycles between −80 °C and room temperature and then stored at −80 °C until analysis. The intracellular accumulation of CML was defined as the fraction of CML in the cells relative to the initial amount of CML in the AP compartment.

### 2.8. Transport Pathway Experiments

#### 2.8.1. Transport Pathway of FC

To investigate whether FC is transported by simple diffusion across Caco-2 monolayers, concentration dependence experiments were conducted in quadruplicate. For AP-BL transport, 0.5 mL HBSS containing FC at three different concentrations was added to the AP compartment, while 1.5 mL HBSS was added to the BL compartment. For BL-AP transport, 1.5 mL HBSS containing FC at three different concentrations was added to the BL compartment, while 0.5 mL HBSS was added to the AP compartment. After 2 h of transport at 37 °C, 0.4 mL samples were collected from the receiver compartment and analyzed for free CML using HPLC-MS/MS.

The transport pathway was evaluated based on concentration dependence and permeability characteristics. If FC transport across Caco-2 monolayers exhibits concentration dependence, simple diffusion was considered the predominant transport pathway [32]. Consistent transport and efflux rates across different concentrations were considered indicative of simple diffusion. Furthermore, *P_app_* values were calculated using Equation (2), and simple diffusion was considered the predominant transport pathway if the *P_app_* values remained constant across the concentration range.

#### 2.8.2. Transport Pathway of BC Digests

##### Transport of BC Digests Using Transport Inhibitors

The transport pathway of BC digests across Caco-2 monolayers was investigated in quadruplicate using a series of transport inhibitors, following the method described by Ding et al. [28]. Differentiated Caco-2 monolayers were pre-incubated for 30 min with 0.5 μg/mL cytochalasin D (a tight junction (TJ) disruptor), 500 nM wortmannin (a transcytosis inhibitor), 10 mM Gly-Pro (a PepT1 substrate), or 10 mM sodium azide (an ATP synthesis inhibitor), added to both the AP and BL compartments. Following pre-incubation, 0.5 mL of HBSS containing BC digests was added to the AP compartment, while 1.5 mL of fresh HBSS was added to the BL compartment. After 2 h of transport, 1 mL samples were collected from the BL compartment and analyzed for total CML content by HPLC-MS/MS. *P_app_* values were subsequently calculated using Equation (2).

##### Effect of BC Digests on Peptide Transporter 1 (PepT1) Expression

To further investigate the involvement of PepT1 in the transport of BC digests, PepT1 expression was analyzed following BC digest treatment. Caco-2 cells were cultured and treated according to the method described by Holik et al. [33]. Caco-2 cells were seeded in 6-well plates at a density of 4.2 × 10^4^ cells/cm^2^ and differentiated for 21 days at 37 °C in a CO_2_ incubator. After washing with preheated PBS, cells were incubated with 2 mL culture medium containing BC digests, while control wells received fresh culture medium only. Following a 24 h incubation, total RNA and protein were extracted from Caco-2 cells. Gene expression was determined by RT-qPCR, and protein expression was analyzed by Western blotting, as previously described by Yuan et al. [16]. Three independent wells were used for each treatment group. The primer sequences used for RT-qPCR analysis are presented in Table S1.

### 2.9. Detection of Free and Total CML by HPLC-MS/MS

Free CML was quantified in samples collected from transport experiments and the transport pathway studies of FC, whereas total CML was detected in samples obtained from transport pathway studies of BC digests. Moreover, both free and total CML contents were measured in samples collected from the intracellular accumulation experiments of FC and BC digests. The analytical procedures for free and total CML determination were adapted from previously established methods [27,34] with slight modifications. For free CML detection, 0.2 mL of 10% TCA solution was added to each sample, followed by incubation on ice for 30 min. The samples were then centrifuged for 10 min at 4 °C, and the resulting supernatants were collected and dried using a rotational vacuum concentrator (Christ RVC 2-18CD plus, Osterode am Harz, Germany). For total CML detection, 0.1 mL of HPLC ultrapure water and 0.5 mL of 0.1 M NaBH_4_ in 0.2 M borate buffer (pH 9.2) were added to each sample. The mixtures were incubated at 20 °C for 2.5 h. Subsequently, 0.6 mL of 12 M HCl was added, and the samples were hydrolyzed under nitrogen at 110 °C for 21 h. After hydrolysis, the samples were dried under a nitrogen stream. Isotopically labeled CML-d_4_ was used as the internal standard and added to each sample before analysis. The remaining sample preparation procedures and the HPLC-MS/MS parameters were employed according to the method of Yuan et al. [27]. The calibration curves, LOD and LOQ for CML are presented in Table S2.

### 2.10. Statistical Analysis

Statistical analyses were conducted using SPSS version 20.0 (IBM Corporation, Armonk, NY, USA), and all data are presented as means ± standard deviation (SD). The normality and homogeneity of variance were assessed using the Shapiro–Wilk test and Levene’s test, respectively. Differences between two groups were evaluated using an unpaired Student’s *t*-test (normal distribution equal variance), Welch‘s corrected *t*-test (normal distribution unequal variance) or Mann–Whitney test (non-normal distribution). For multiple comparisons, a one-way analysis of variance (ANOVA) followed by Tukey’s post hoc test was used for normally distributed data, whereas a Kruskal–Wallis test followed by Dunn’s test was applied to non-parametric data. Statistical significance was set at *p* < 0.05.

## 3. Results and Discussion

### 3.1. Effect of Free CML and BSA-CML Digests on Cytotoxicity of Caco-2 Cells

**Figure 1:**
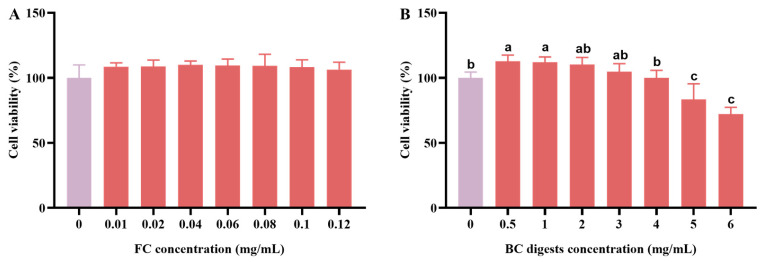
Effect of FC (**A**) and BC digests (**B**) at various concentrations on viability of differentiated Caco-2 cells after 24 h of exposure. Cell viability was determined using CCK-8 assay. Data are presented as mean ± SD (*n* = 6 biological replicates). Different letters indicate significant differences according to one-way ANOVA followed by Tukey’s post hoc test (*p* < 0.05).

Prior to transepithelial transport experiments, the potential cytotoxic effects of FC and BC digests on Caco-2 cells were evaluated using the CCK-8 assay to determine appropriate exposure concentrations. As presented in Figure 1A, treatment with FC at concentrations ranging from 0.01 to 0.12 mg/mL did not significantly affect Caco-2 cell viability compared with the untreated control group (*p* > 0.05). As illustrated in Figure 1B, exposure to BC digests at concentrations between 2 and 4 mg/mL resulted in no significant reduction in cell viability compared with the control group (*p* > 0.05) (Figure 1B).

To ensure sufficient analytical sensitivity while maintaining physiological relevance, BC digests (4 mg/mL) were selected for subsequent transport experiments. At this concentration, the corresponding CML concentration in the BC digests was 0.1 mg/mL. Therefore, FC was also applied at a CML-equivalent concentration of 0.1 mg/mL to enable a direct comparison of transport behaviors between the free and protein-bound forms of CML. The selected concentration was further evaluated for its physiological relevance. Based on reported dietary CML intake (0.06–0.07 mg/kg BW/day) and high-exposure scenarios (0.16–0.24 mg/kg BW/day) [3], together with the typical postprandial gastric volume of approximately 0.5–1 L [35], the intestinal concentration of dietary CML was estimated to be approximately 0.034 mg/mL. Although lower than the concentration used in the present study, the selected level remained within the same order of magnitude and therefore represents a physiologically relevant exposure scenario. Furthermore, the experimental concentration employed in this study was consistent with that reported by Wu et al. [36], who used 0.5 mM CML (approximately 0.1 mg/mL), thereby facilitating direct comparison with previous studies.

### 3.2. Transepithelial Transport of Free CML and BSA-CML Digests

#### 3.2.1. Characterization of Caco-2 Cell Monolayer Model

##### TEER

**Figure 2:**
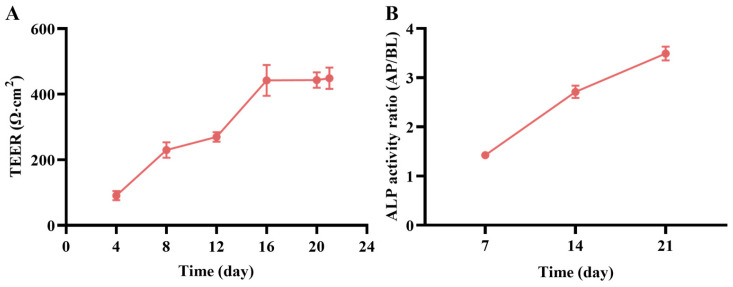
Trends in TEER (**A**) and ALP activity ratio (AP/BL) (**B**) in Caco-2 cell monolayer during model establishment. Data are presented as mean ± SD (*n* = 3 biological replicates).

TEER is widely recognized as an indicator of cell confluence, monolayer integrity and TJ formation in Caco-2 cell models. A TEER value exceeding 300 Ω·cm^2^ is generally considered indicative of a functionally intact monolayer suitable for intestinal transport studies [28]. As shown in Figure 2A, the TEER values increased rapidly between days 4 and 16, reaching 442 ± 47 Ω·cm^2^ on day 16. Thereafter, TEER values remained relatively stable until day 21, maintaining values around 440 Ω·cm^2^. These results suggest that the Caco-2 cells had undergone sufficient differentiation and formed an intact monolayer structure by day 21.

##### ALP Activity

ALP is a characteristic brush border enzyme of intestinal epithelial cells and is widely used as a biochemical marker of Caco-2 monolayer differentiation [37]. To further evaluate the maturation and polarization of the monolayers, ALP activity was determined in the AP and BL compartments on days 7, 14, and 21. As depicted in Figure 2B, the ALP activity ratio increased progressively from 1.43 ± 0.07 on day 7 to 3.49 ± 0.14 on day 21. This gradual increase indicates the preferential localization of ALP on the AP side of the Caco-2 monolayer, which is characteristic of polarized intestinal epithelial cells. The marked enrichment in ALP activity on the AP side therefore confirms the development of enterocyte-like polarization and complete differentiation of the Caco-2 monolayer.

##### Sodium Fluorescein Permeability

While TEER and ALP measurements evaluate monolayer integrity and differentiation, the assessment of paracellular permeability provides complementary information regarding barrier function. Sodium fluorescein is a well-established paracellular permeability marker and is commonly used to evaluate the tightness of epithelial monolayers [38]. In the present study, the *P_app_* of sodium fluorescein in the AP-BL direction was (0.41 ± 0.03) × 10^−6^ cm/s. This value was lower than the generally accepted threshold of 0.5 × 10^−6^ cm/s for intact Caco-2 monolayers, indicating limited paracellular leakage. Together, the TEER, ALP, and sodium fluorescein permeability results demonstrated that the established Caco-2 monolayers possessed appropriate integrity, differentiation, and barrier function and were suitable for subsequent transport experiments.

#### 3.2.2. Transepithelial Transport of Free CML and BSA-CML Digests

**Figure 3:**
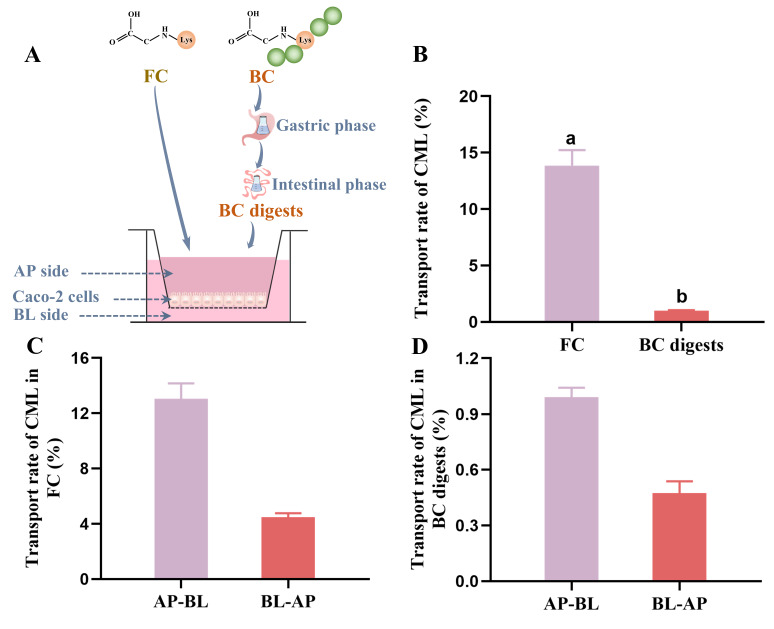
Bidirectional transepithelial transport of FC and BC digests across Caco-2 monolayers after 2 h incubation. (**A**) Schematic illustration of transport experiment. (**B**) AP-to-BL transport rates of CML from FC and BC digests. (**C**) Bidirectional transport of FC-derived CML. (**D**) Bidirectional transport of CML from BC digests. Data are presented as mean ± SD (*n* = 4 biological replicates). For panel (**B**), different letters indicate significant differences between groups determined by an unpaired Student’s *t*-test (*p* < 0.05).

A previous study investigated the transport of free CML and gastrointestinal digests of glyoxal (GO) glycated BSA across Caco-2 monolayers [36]. However, differences in the CML exposure concentration limited direct comparisons of transport efficiency, and the underlying transport pathways were not explored. To directly compare the transepithelial transport of free and protein-bound CML under equivalent exposure conditions, FC and BC digests containing the same CML content were applied to Caco-2 monolayers, and the amount of transported CML was quantified after 2 h (Figure 3A). As depicted in Figure 3B, the transport rate of CML from FC reached 13.04% ± 1.12%, which was significantly higher than that from BC digests (0.99% ± 0.05%) (*p* < 0.05). This finding is consistent with our previous pharmacokinetic observations in rats [19], thereby supporting our original hypothesis that the chemical form of CML influences its intestinal transport behavior. Previous studies reported a transport rate of approximately 18% for free CML after 3 h of transport across Caco-2 monolayers [36], whereas gastrointestinal digests of GO glycated BSA and glycated myofibrillar proteins exhibited transport rates of 25.95% (3 h) and 35.06% (2 h), respectively [23,36]. In the present study, the transport rate of FC was comparable to that previously reported for free CML, whereas the transport rate of BC digests was lower than the values reported for other glycated protein systems. These differences are likely attributed to substantial variations in glycated substrates, digestion procedures, and transport durations between our study and previous reports, thereby limiting direct quantitative comparisons [39,40].

To further evaluate apparent transepithelial permeability, *P_app_* was calculated (Table 1). *P_app_* is widely used as an in vitro indicator of epithelial permeability and serves as a useful indicator for comparing the transport characteristics of compounds across Caco-2 monolayers [41]. Previous studies have suggested that compounds with *P_app_* values below 1 × 10^−6^ cm/s generally exhibit low permeability, whereas those with *P_app_* values between 1 and 10 × 10^−6^ cm/s exhibit moderate permeability in the Caco-2 monolayer model [42]. The *P_app_* value of FC-derived CML was (8.09 ± 0.69) × 10^−6^ cm/s, indicating moderate apparent permeability across the Caco-2 monolayer. In contrast, the *P_app_* value of CML from BC digests was only (0.61 ± 0.03) × 10^−6^ cm/s, indicating low apparent permeability. Moreover, the *P_app_* value of FC-derived CML was significantly higher than that of BC digest-derived CML (*p* < 0.05), further demonstrating that the free form of CML exhibited substantially greater apparent transepithelial permeability. It should be noted that the Caco-2 model lacks several physiological features of the native intestine, including mucus secretion, villus architecture, luminal flow, and systemic circulation. Therefore, the *P_app_* values reported here should be interpreted as comparative indicators of transepithelial permeability rather than direct predictors of human intestinal absorption.

Considering the potential efflux transport of CML from myofibrillar protein-bound CML hydrolysates [23], bidirectional transport experiments were performed to distinguish absorptive and secretory transport. As shown in Figure 3C,D, the AP-to-BL transport rates of FC and BC digests were 13.04% ± 1.12% and 0.99 ± 0.05%, respectively, whereas BL-to-AP transport rates were 4.48% ± 0.28% and 0.47% ± 0.06%, respectively. In both treatment groups, absorptive transport exceeded secretory transport. These results indicate that both FC and BC digests underwent net transepithelial transport across Caco-2 monolayers, with FC exhibiting substantially greater transport efficiency than BC digests.

### 3.3. Intracellular Accumulation of Free CML and BSA-CML Digests

**Figure 4:**
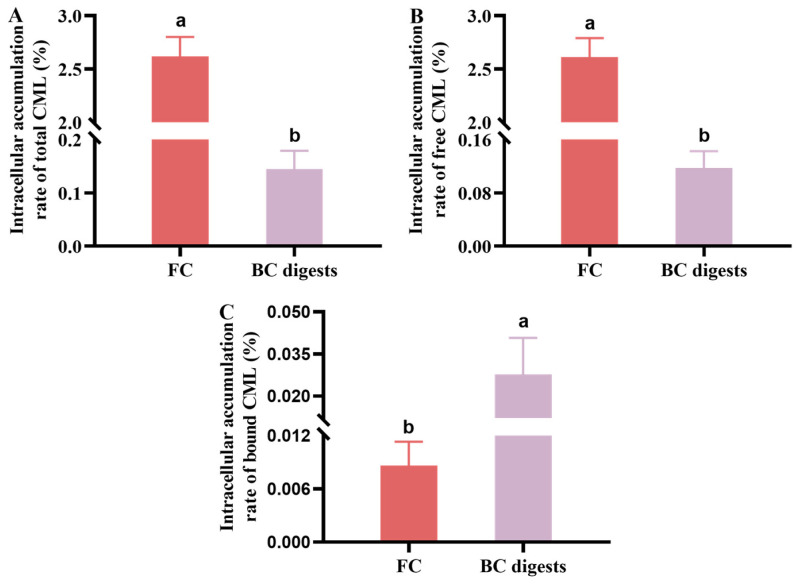
Intracellular accumulation of total CML (**A**), free CML (**B**) and bound CML (**C**) in Caco-2 cell monolayers after 2 h exposure to FC or BC digests. Data are presented as mean ± SD (*n* = 4 biological replicates). Different letters indicate significant differences between groups determined by an unpaired Student’s *t*-test (*p* < 0.05).

To further investigate cellular uptake during transepithelial transport, intracellular CML accumulation was quantified in Caco-2 monolayers after exposure to FC and BC digests. As shown in Figure 4A, the intracellular accumulation of total CML reached 2.62% in the FC group, which was significantly higher than that observed in the BC digest group (0.14%) (*p* < 0.05). This result indicates that CML in its free form is more readily taken up by intestinal epithelial cells than CML derived from BC digests. The accumulation of CML in intestinal tissues was also reported in our previous pharmacokinetic study [19]. Similarly, earlier Caco-2 transport studies reported the intracellular accumulation of CML following exposure to free CML and glycated β-casein digests [35,40]. In the present study, the intracellular accumulation rates of free CML derived from FC and BC digests were 2.61% ± 0.18% and 0.12% ± 0.03%, respectively (Figure 4B). Notably, the intracellular accumulation rate of free CML in the FC-treated group (2.61%) was comparable to that reported previously (approximately 3.5%) [35], further supporting the reliability of the current results.

Upon calculating the amount of bound CML, it was observed that bound CML accounted for 0.03% and 0.01% of the initial CML dose in the BC digest and FC groups, respectively (Figure 4C). Because only trace amounts of bound CML were detected in the FC-treated cells, we hypothesized that this signal originated from endogenous bound CML already present in the Caco-2 monolayers rather than from FC uptake. To verify this hypothesis, the amount of bound CML was determined in untreated (blank) Caco-2 cell monolayers. The intracellular bound CML level in the blank control group was 3.63 ± 0.58 ng/well, compared with 4.35 ± 1.36 ng/well in the FC group, with no significant difference between the two groups (*p* > 0.05). These results indicate that the small amount of bound CML detected following FC treatment primarily reflected the endogenous cellular background. In contrast, treatment with BC digests significantly increased intracellular bound CML levels to 13.31 ± 3.49 ng/well (*p* < 0.05 versus blank control), demonstrating that bound CML derived from BC digests was taken up by the cells. Taken together, the intracellular accumulation of total CML in the FC group was almost entirely attributable to free CML uptake, whereas BC digests contributed both free and bound CML to the intracellular pool. Nevertheless, the overall accumulation of CML was substantially lower in the BC digest group than in the FC group. Combined with the transepithelial transport results, these findings suggest that protein-bound or peptide-bound CML exhibits markedly lower intestinal uptake efficiency than free CML under the experimental conditions employed in this study.

### 3.4. Transport Pathway of Free CML and BSA-CML Digests

#### 3.4.1. Transport Pathway of Free CML

**Figure 5:**
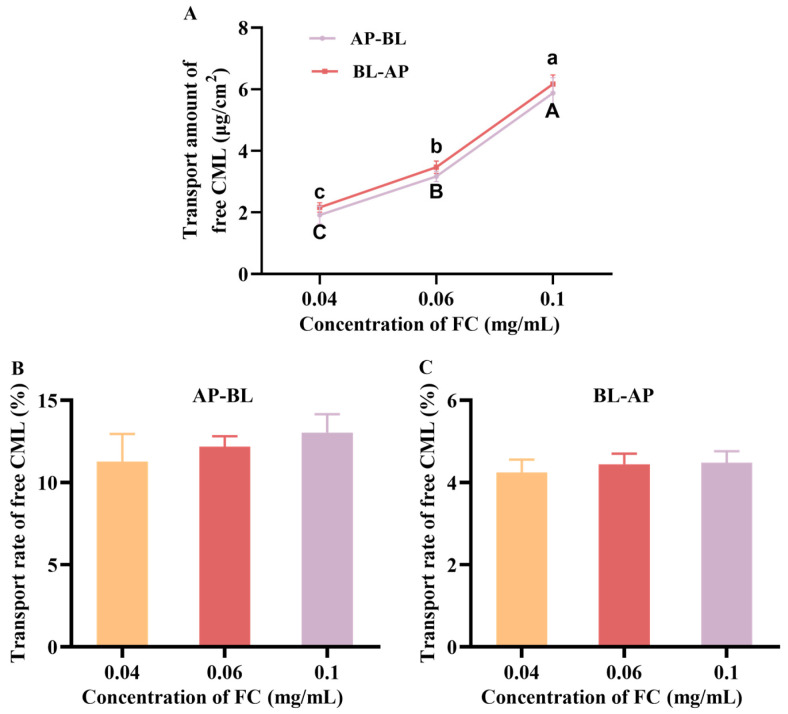
Concentration-dependent transepithelial transport of FC across Caco-2 cell monolayers. (**A**) Amount of transported free CML. (**B**) Transport rate of free CML (AP-BL). (**C**) Transport rate of free CML (BL-AP). Data are presented as mean ± SD (*n* = 4 biological replicates). Different uppercase letters (AP-BL) and lowercase letters (BL-AP) indicate significant differences among groups according to one-way ANOVA followed by Tukey’s post hoc test (*p* < 0.05).

Due to the low affinity of free CML for the intestinal lysine transporter during transepithelial transport across Caco-2 monolayers, Hellwig et al. [22] proposed that free CML is primarily transported via simple diffusion rather than active transport. To verify this hypothesis, FC was applied at three different concentrations, and its transport behavior across Caco-2 cell monolayers was evaluated to determine whether transport was concentration-dependent. As shown in Figure 5A, the amount of free CML transported in both the AP-BL and BL-AP directions increased with growing FC concentration (0.04–0.10 mg/mL) and did not exhibit saturation within the tested range. This concentration-dependent transport profile is characteristic of simple diffusion. Consistent with this observation, the AP-BL transport rates at FC concentrations of 0.04, 0.06, and 0.1 mg/mL were 11.28% ± 1.68%, 12.18% ± 0.64% and 13.04% ± 1.12%, respectively, with no significant differences among groups (*p* > 0.05) (Figure 5B). Similarly, the corresponding BL-AP transport rates were 4.25% ± 0.31%, 4.45% ± 0.26% and 4.48% ± 0.28%, respectively, and likewise showed no significant differences (*p* > 0.05) (Figure 5C). The relatively constant transport and efflux rates suggested that FC transport appeared to occur mainly through simple diffusion.

*P_app_* values were further calculated for FC at different concentrations during transepithelial transport across Caco-2 cell monolayers over a 2 h period. The *P_app_* (AP-BL) values ranged from 6.99 × 10^−6^ to 8.09 × 10^−6^ cm/s, with no significant differences among concentrations (*p* > 0.05) (Table 2). The concentration-independent *P_app_* values indicated that FC transport occurs via simple diffusion. Moreover, the ratio of bidirectional permeability coefficients has been widely used to identify active transport processes, with values close to unity indicating simple diffusion [43]. In the present study, the ratios of *P_app_* (AP-BL) to *P_app_* (BL-AP) were 1.15, 1.10 and 1.04 at FC concentrations of 0.04, 0.06, and 0.1 mg/mL, respectively (Table 2), further supporting simple diffusion as the dominant transport pathway for FC.

#### 3.4.2. Transport Pathway of BSA-CML Digests

**Figure 6:**
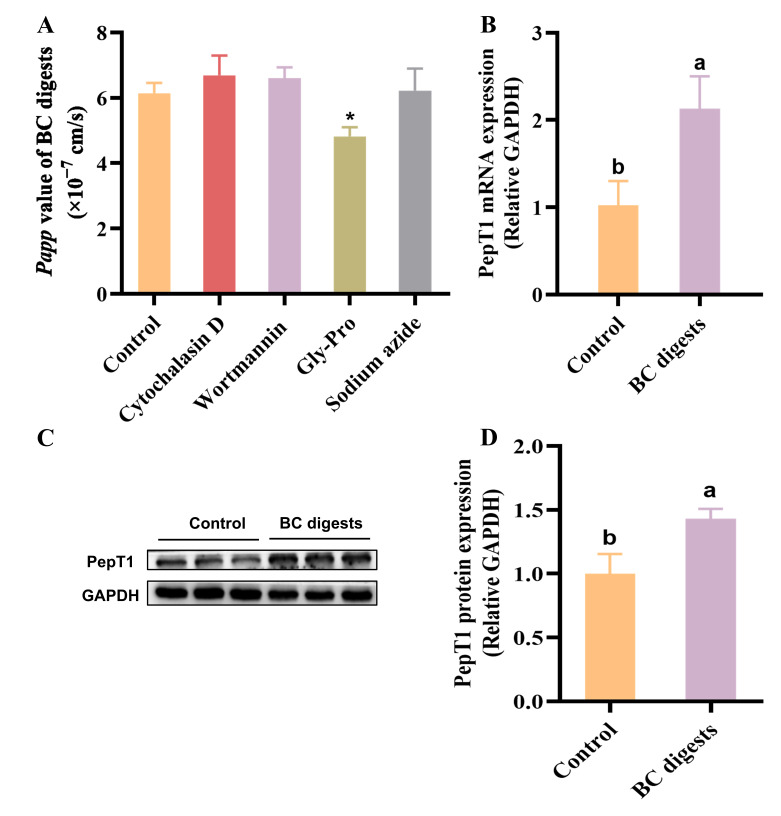
Transport pathway of BC digests in Caco-2 cell monolayers. (**A**) Effects of transport inhibitors on *P_app_* value of CML from BC digests. (**B**) Relative PepT1 mRNA expression. (**C**) Representative Western blot images of PepT1. (**D**) Relative PepT1 protein expression. Data are presented as mean ± SD (*n* = 4 biological replicates for transport inhibitor experiments; *n* = 3 biological replicates for RT-qPCR and Western blot analyses). * Asterisks indicate significant differences compared with control group following one-way ANOVA with Tukey’s post hoc test (*p* < 0.05). Different letters indicate significant differences between groups determined by unpaired Student’s *t*-test (*p* < 0.05).

Besides the minimal amounts of free and protein-bound CML, peptide-bound CML was the predominant form present in the BC digests. Peptide-bound CML, predominantly associated with small peptides < 5 kDa, accounted for 94.7% of the total CML in BC digests [27]. To identify the pathway responsible for the transepithelial transport of CML derived from BC digests, a series of inhibitors was employed. Cytochalasin D, a TJ disruptor known to alter cytoskeletal structure and modulate paracellular permeability [44], was used to evaluate the involvement of the paracellular route. As shown in Figure 6A, cytochalasin D treatment did not significantly affect the transport of CML from BC digests, indicating that passive paracellular transport through TJs was unlikely to contribute substantially to CML absorption. To investigate the role of transcytosis, wortmannin, an irreversible inhibitor of phosphatidylinositol-3-kinase, was employed [45]. No significant change in CML transport was observed following wortmannin treatment (Figure 6A), suggesting that transcytosis was not a major transport pathway for CML derived from BC digests. In contrast, treatment with Gly-Pro, a competitive substrate of PepT1, was used to examine whether the transepithelial transport of the peptide occurs via carrier-mediated pathways [46]. The transport of CML from BC digests significantly decreased (*p* < 0.05) after Gly-Pro treatment, suggesting the involvement of PepT1 in the transepithelial transport of CML derived from BC digests (Figure 6A). To further explore this possibility, PepT1 expression at both the mRNA and protein levels was subsequently evaluated.

As shown in Figure 6B–D, treatment with BC digests significantly upregulated both PepT1 mRNA and protein expression compared with the control group (*p* < 0.05). The combined evidence from competitive inhibition and transporter upregulation provides support for the participation of PepT1 in the transepithelial transport of CML derived from BC digests. Sodium azide, an inhibitor of ATP synthesis, was used to assess energy dependence [46]. Pre-incubation with sodium azide did not result in a significant decrease in CML transport from BC digests (*p* > 0.05) (Figure 6A). Because PepT1 is driven by a transmembrane proton electrochemical gradient rather than directly by ATP hydrolysis [22], the absence of a sodium azide effect does not necessarily exclude the involvement of PepT1 under the present experimental conditions. Therefore, the proposed involvement of a PepT1-associated transport pathway is supported by the combined evidence of Gly-Pro competitive inhibition and PepT1 upregulation, rather than the sodium azide experiment alone. Further studies using more specific approaches to perturb proton-coupled transport will be required to clarify the energy dependence of this transport process. Collectively, these findings support the involvement of a PepT1-associated pathway in the transepithelial transport of CML derived from BC digests. This conclusion is consistent with previous findings showing that CML-containing hydrolysates derived from glycated myofibrillar protein were transported via a PepT1-associated pathway [23]. Similarly, several studies have shown that peptide-bound pyrraline and CML, particularly when associated with dipeptides and tripeptides, are efficiently transported via PepT1 [30,40,47,48]. Furthermore, Newstead et al. [49] reported that PepT1 preferentially recognizes dipeptide and tripeptide residues, whereas longer peptides generally exhibit poor affinity for this transporter. Considering that peptide-bound CML constituted the predominant form of CML in BC digests and that PepT1 preferentially transports dipeptides and tripeptides, it is reasonable to hypothesize that dipeptide- and tripeptide-bound CML may be involved in the observed PepT1-associated transport.

Interestingly, Zhao et al. [21] mentioned that peptide-bound AGEs may exhibit greater intestinal absorption than free AGEs, as carrier-mediated transport is generally more efficient than simple diffusion. However, the present study demonstrated the substantially lower transport of BC digest-derived CML than FC-derived CML. One possible explanation is that only a limited proportion of CML in the BC digests was present as PepT1-recognizable dipeptide and tripeptide species, thereby restricting overall transport efficiency. However, a detailed structural characterization of the BC digests was not performed in the present study. Consequently, the specific dipeptide- and tripeptide-bound CML species responsible for PepT1 recognition remain unidentified, limiting a more precise interpretation of the proposed transport pathway. Future studies employing peptidomic characterization will help identify the specific CML-modified peptide species involved in PepT1-associated transport. In addition, TEER was assessed only at the completion of the transport experiments rather than immediately after inhibitor treatment. Although no significant reduction in TEER was observed after the 2 h transport period, transient changes in monolayer integrity during inhibitor exposure cannot be completely excluded. Future studies incorporating TEER measurements both before and after inhibitor treatment, together with complementary barrier integrity assays, would further strengthen the interpretation. Moreover, all transport experiments were conducted using a single incubation time (2 h). Therefore, the present results mainly reflect transport behavior at this time point rather than the complete transport kinetics. Studies incorporating multiple sampling time points will be required to further characterize the transport kinetics of different forms of dietary CML. Furthermore, a complete mass balance analysis of CML, including the quantification of the donor, receiver, intracellular, and unrecovered fractions, was not performed in the present study. Although the current results allow for a comparison of the relative transepithelial transport behaviors of FC and BC digests, future studies incorporating comprehensive mass balance analysis will further strengthen the quantitative evaluation of CML transport across intestinal epithelial cells. Together, these future studies will strengthen the mechanistic understanding and quantitative evaluation of the intestinal transport of different forms of dietary CML.

## 4. Conclusions

This study compared the transepithelial transport, intracellular accumulation, and transport pathways of FC and BC digests using a Caco-2 cell monolayer model. The results indicated that FC exhibited significantly greater transepithelial transport and higher apparent permeability than BC digests, as evidenced by higher transport rates (13.04% vs. 0.99%), greater *P_app_* values, and the increased intracellular accumulation of CML. These findings are consistent with our previous pharmacokinetic observations and further highlight that the chemical form of dietary CML markedly influences its intestinal transport behavior. Notably, FC appeared to be transported predominantly via simple diffusion, whereas the transepithelial transport of CML derived from BC digests likely involved a PepT1-associated pathway. Despite the likely involvement of PepT1-associated transport, BC digests exhibited substantially lower overall transport efficiency than FC under the experimental conditions employed. This difference may be attributed to the distinct molecular forms of CML present in the two treatments, with FC existing entirely as free CML and BC digests consisting predominantly of peptide-bound CML. Overall, the present study provides valuable insights into the intestinal transport behavior of free and protein-bound CML. These findings contribute to a better understanding of the gastrointestinal fate of dietary AGEs and provide a basis for evaluating their potential health effects.

## Figures and Tables

**Table 1:** *P_app_* value of CML in FC and BC digests during transport for 2 h (AP-BL).

Sample	*P_app_* (×10^−6^ cm/s)
FC	8.09 ± 0.69 a
BC digests	0.61 ± 0.03 b

Data are presented as mean ± SD, *n* = 4. Different letters indicate significant differences between groups determined by unpaired Student’s *t*-test (*p* < 0.05).

**Table 2:** *P_app_* value of FC at different concentrations during transepithelial transport across Caco-2 cell monolayers for 2 h.

FC Concentration (mg/mL)	*P_app_* (×10^−6^ cm/s) (AP-BL)	*P_app_* (×10^−6^ cm/s) (BL-AP)	*P_app_* (BL-AP)/*P_app_* (AP-BL)
0.04	6.99 ± 1.04	7.90 ± 0.57	1.15 ± 0.17
0.06	7.55 ± 0.40	8.27 ± 0.48	1.10 ± 0.06
0.1	8.09 ± 0.69	8.33 ± 0.53	1.04 ± 0.09

Data are presented as mean ± SD, *n* = 4. Statistical analysis was performed using one-way ANOVA followed by Tukey’s post hoc test. No significant differences were observed among three concentrations (*p* > 0.05).

## Data Availability

The original contributions presented in this study are included in the article/Supplementary Materials. Further inquiries can be directed to the corresponding authors.

## Supplementary Materials

The following supporting information can be downloaded at https://www.mdpi.com/article/10.3390/foods15142432/s1, Table S1: The genes and primer sequences for RT-qPCR; Table S2: Summary of analytical performance parameters of CML determination.

## Abbreviations

The following abbreviations are used in this manuscript:

AGEs	Advanced glycation end products
CML	Nε-carboxymethyllysine
FC	Free CML
BSA-CML	Bovine serum albumin–CML
BC	Bound CML
PepT1	Peptide transporter 1
Nrf2	Nuclear factor E2-related factor 2
NF-κB	Nuclear factor kappa-B
FBS	Fetal bovine serum
DMEM	Dulbecco’s modified Eagle medium
EDTA	Ethylenediaminetetraacetic acid
HBSS	Hanks’ balanced salt solution
CCK-8	Cell Counting Kit-8
PET	Transwell polyester
AP	Apical
BL	Basolateral
TEER	Transepithelial electric resistance
ALP	Alkaline phosphatase
Papp	Apparent permeability coefficient
TJs	Tight junctions
HPLC-MS/MS	High-performance liquid chromatography–mass spectrometry
SD	Standard deviation
ANOVA	One-way analysis of variance
