# Supplementary material for: The Transepithelial Transport and Transport Pathways of Free and Bound Nε-Carboxymethyllysine Using a Caco-2 Cell Monolayer Model
Source: Foods. 2026 Jul 9;15(14):2432. doi: 10.3390/foods15142432 (PMC13408533; doi:10.3390/foods15142432)
Supplement: Supplementary file 1 [file foods-15-02432-s001.zip › foods-4385467-supplementary.pdf]

Supplementary information

Table S1 The genes and primer sequences for RT-qPCR

Genes	Forward Primer（5'-3'）	Reverse Primer（5'-3'）
PepT1	TCTTTGGTTATCCCCTGAGCA	GGCGGTGGACAGGTTATCATC
GAPDH	GGAGCGAGATCCCTCCAAAAT	GGCTGTTGTCATACTTCTCATGG

Table S2 Summary of the analytical performance parameters of the CML determination

analyte	linearity			sensitivity	
	Linear equations	linearity range (ng/mL)	R^2	LOD (ng/mL)	LOQ (ng/mL)
CML	y=1.0752x-0.0298	1-2500	0.9994	0.361	1.202
